# Functional Inactivation of *Drosophila* *GCK* Orthologs Causes Genomic Instability and Oxidative Stress in a Fly Model of MODY-2

**DOI:** 10.3390/ijms22020918

**Published:** 2021-01-18

**Authors:** Elisa Mascolo, Francesco Liguori, Lorenzo Stufera Mecarelli, Noemi Amoroso, Chiara Merigliano, Susanna Amadio, Cinzia Volonté, Roberto Contestabile, Angela Tramonti, Fiammetta Vernì

**Affiliations:** 1 Department of Biology and Biotechnology “Charles Darwin”, Sapienza University, 00185 Rome, Italy; Elisa.Mascolo@uniroma1.it (E.M.); stuferamecarelli.1752614@studenti.uniroma1.it (L.S.M.); amorosonoemi1996@gmail.com (N.A.); meriglia@usc.edu (C.M.); 2 Preclinical Neuroscience, IRCCS Santa Lucia Foundation, 00143 Rome, Italy; f.liguori@hsantalucia.it (F.L.); s.amadio@hsantalucia.it (S.A.); cinzia.volonte@cnr.it (C.V.); 3 Department of Molecular and Computational Biology, University of Southern California, Los Angeles, CA 90089, USA; 4 Institute for Systems Analysis and Computer Science “A. Ruberti”, National Research Council (IASI-CNR), 00185 Rome, Italy; 5 Istituto Pasteur Italia-Fondazione Cenci Bolognetti and Department of Biochemical Sciences “A. Rossi Fanelli”, Sapienza University, 00185 Rome, Italy; roberto.contestabile@uniroma1.it (R.C.); angela.tramonti@cnr.it (A.T.); 6 Istituto di Biologia e Patologia Molecolari, Consiglio Nazionale delle Ricerche, 00185 Rome, Italy

**Keywords:** MODY-2, *Drosophila melanogaster*, glucokinase, chromosome aberrations, vitamin B6

## Abstract

Maturity-onset diabetes of the young (MODY) type 2 is caused by heterozygous inactivating mutations in the gene encoding glucokinase (GCK), a pivotal enzyme for glucose homeostasis. In the pancreas GCK regulates insulin secretion, while in the liver it promotes glucose utilization and storage. We showed that silencing the *Drosophila* *GCK* orthologs *Hex-A* and *Hex-C* results in a MODY-2-like hyperglycemia. Targeted knock-down revealed that *Hex-A* is expressed in insulin producing cells (IPCs) whereas *Hex-C* is specifically expressed in the fat body. We showed that *Hex-A* is essential for insulin secretion and it is required for *Hex-C* expression. Reduced levels of either Hex-A or Hex-C resulted in chromosome aberrations (CABs), together with an increased production of advanced glycation end-products (AGEs) and reactive oxygen species (ROS). This result suggests that CABs, in GCK depleted cells, are likely due to hyperglycemia, which produces oxidative stress through AGE metabolism. In agreement with this hypothesis, treating GCK-depleted larvae with the antioxidant vitamin B6 rescued CABs, whereas the treatment with a B6 inhibitor enhanced genomic instability. Although MODY-2 rarely produces complications, our data revealed the possibility that MODY-2 impacts genome integrity.

## 1. Introduction

Maturity-onset diabetes of the young (MODY) is a heterogeneous group of disorders due to mutations in single genes involved in insulin metabolism. It is inherited as an autosomal dominant trait and represents 2–5% of diabetes cases [1,2]. To date, 14 subtypes of MODY, different in terms of gene mutation, age at onset and pattern of hyperglycemia, have been identified [3]. MODY-2 or GCK-MODY is one of the most common subtypes, caused by heterozygous inactivating mutations in the gene encoding the glucokinase enzyme (GCK), a key player in glucose homeostasis maintenance [4,5]. GCK catalyzes the conversion of glucose to glucose-6-phosphate and presents distinctive features, such as low affinity for glucose, moderate cooperative binding of substrate, and lack of significant feedback inhibition [6]. *GCK* is expressed in pancreatic β-cells, in liver, and in few brain and gastrointestinal neuroendocrine cells [6]. This differential expression is explained by the presence of two tissue-specific promoters, leading to the production of transcripts different only for their first exon size [7,8]. In pancreatic islets, GCK functions as a glucose sensor [6,7,9,10] and regulates insulin secretion in response to blood glucose levels [9,10]. In neuroendocrine cells, GCK acts similarly to regulate the responses of these tissues to changes in blood glucose levels [6,11]. In the hepatocytes, GCK determines the rate of both glucose uptake and glycogen synthesis and is essential for the regulation of various glucose-responsive genes [12].

In hepatocytes, *GCK* expression is regulated at both transcriptional and post-translational levels [13]. In the liver, hormones and glucose metabolites modulate *GCK* transcription, thus explaining the immediate response to the nutritional state [7]. Insulin induces *GCK* transcription by acting through several pathways, such as phosphoinositide-3 (PI-3)-kinase/protein kinase B (PKB) and mitogen-activated protein (MAP)-kinase pathways [14].

Post-translationally, GCK is negatively regulated by the reversible binding to glucokinase regulatory protein (GKRP), which sequesters GCK into the nucleus, preventing its ability to participate in cytosolic glycolysis; conversely, GCK is activated and stabilized by 6-phosphofructo-2-kinase/fructose 2,6-bisphosphatase (PFK2/FBP2) [15].

In pancreatic islets, *GCK* is not transcriptionally regulated, given that the glucose-sensing mechanism of these cells requires a constant level of the enzyme. Indeed, glucose is the main GCK activator, and immediately amplifies its activity by a cooperative effect. There is evidence that in the pancreas GCK interacts with insulin secretory granules, although the function of this association is still debated [16,17].

In agreement with the roles played by GCK in liver and pancreas, GCK-MODY is characterized by a mild diabetes (5,5–8,0 mmol/L) with signs of pancreatic β-cell and hepatocyte dysfunctions [18]. Reduced GCK activity in pancreatic β-cells increases the glucose threshold for insulin secretion, resulting in a fasting hyperglycemia [19,20]. Reduced GCK activity in the liver is, instead, responsible for decreased glycogen accumulation and increased hepatic gluconeogenesis, contributing to the post-prandial hyperglycemia observed in patients with MODY-2 [21].

The majority of individuals with *GCK* mutations have normal insulin sensitivity, but moderate insulin resistance can be developed over time [22]. Usually, patients with MODY-2 are not insulin-treated, except in the case of pregnant women not passing their *GCK* mutation to the fetus [23]. In addition, some commonly used hypoglycemic drugs have often been reported as ineffective on GCK-MODY [24]. Patients with MODY-2 present lipid and blood pressure levels similar to the general population, thus hyperglycemia represents an isolated risk factor for complications in patients with *GCK* mutations [18,25,26].

The *GCK* gene is evolutionarily conserved across different species, and studies carried out in different model organisms highly contributed to the clarification of GCK-MODY pathophysiology [6,7]. Complex molecular and genetic strategies have been applied to specifically inactivate either the hepatic or the pancreatic isoform in mice in order to better understand the role of GCK in specific tissues [27,28,29,30]. In addition, mutagenesis screening carried out in mice led to the isolation of several *GCK* mutations, also found in human patients, so paving the way to personalized care [31,32,33,34].

In the last decade, *Drosophila* turned out to be a great model system for metabolism-related diseases. Recent studies have indeed revealed a high degree of conservation of major cellular metabolism pathways between humans and *Drosophila* [35,36,37]. The *Drosophila* genome harbors four different GCK orthologs: *Hex-A*, *Hex-C*, *Hex-t1,* and *Hex-t2*, still poorly characterized. It has been shown that silencing either *Hex-A* (CG3001) or *Hex-C* (CG8094) genes prevents larval-to-pupal metamorphosis, when larvae were reared in a sucrose rich medium, thus suggesting that both genes could be involved in glucose metabolism [38]. A subsequent study showed that *Hex-C* is expressed in the fat body, the *Drosophila* counterpart of mammalian liver and adipose tissue, but not in insulin producing cells (IPCs), functionally corresponding to the pancreas. It is therefore unknown which fly gene might play the role of pancreatic glucose sensor [39]. In addition, it has been reported that, analogously to what occurs in mammals, the expression of *Hex-C* in the fat body is regulated by the nuclear factor HFN4-α (encoded by the MODY-1 associated gene), suggesting that, despite the presence of different genes for GCK function in *Drosophila*, basic patterns for the modulation of this enzyme are evolutionarily conserved [39].

In this paper we demonstrated that *Hex-A* is expressed in *Drosophila* IPCs and that it is involved in insulin secretion, thus mimicking the mammalian *GCK* pancreatic isoform. We found that a 55% reduction of *Hex-A* transcript produces a typical MODY-2 phenotype. We also showed that *Hex-A* as well as *Hex-C* downregulation induces chromosome damage mainly caused by reactive oxygen species (ROS) production triggered by advanced glycation end-products (AGEs). Overall, these studies revealed that a reduced GCK activity determines oxidative stress, as already observed in other common forms of diabetes.

## 2. Results

### 2.1. Tissue Specific Silencing of Two Orthologs of Mammalian GCK Causes MODY-2 in Drosophila

In the course of a study aimed at investigating the relationship between hyperglycemia and DNA damage in *Drosophila*, we found that the silencing of two *GCK* orthologs (*Hex-A* and *Hex-C*) caused chromosome aberrations (CABs). Before studying the involvement of these two genes in DNA damage, we explored their role in glucose homeostasis. In mammals, the GCK enzyme is encoded by a single gene, but multiple promoters and alternative splicing yield tissue-specific isoforms. The GCK isoform expressed in pancreatic β-cells acts as a glucose sensor and promotes insulin secretion; the hepatic isoform is required in the liver for glucose uptake and its conversion into glycogen [6].

In *Drosophila*, GCK is encoded by four different genes: *Hex-A*, *Hex-C*, *Hex-t1,* and *Hex-t2*, however, so far only *Hex-C (CG8094)* has been partly characterized. Barry and Thummel demonstrated that the silencing of *Hex-C* by RNA interference produces a MODY-2-like hyperglycemia. However, Hex-C-depleted flies cannot fully recapitulate this disease because *Hex-C* is specifically expressed in the fat body, but not in IPCs, raising the question of which enzyme mimics the pancreatic GCK function in *Drosophila* [39].

We hypothesized that *Hex-A* could be a good candidate for the role of glucose sensor in IPCs because silencing of this gene, as well as silencing of *Hex-C*, produced a high rate of sucrose-induced lethality, in contrast to *Hex-t1* and *Hex-t2*-depleted larvae, in which sucrose does not impact or has a mild impact on vitality, respectively [38]. These data prompted us to think that *Hex-A* and *Hex-C* were more strongly involved in carbohydrate metabolism with respect to the other two *GCK* orthologs.

**Figure 1:**
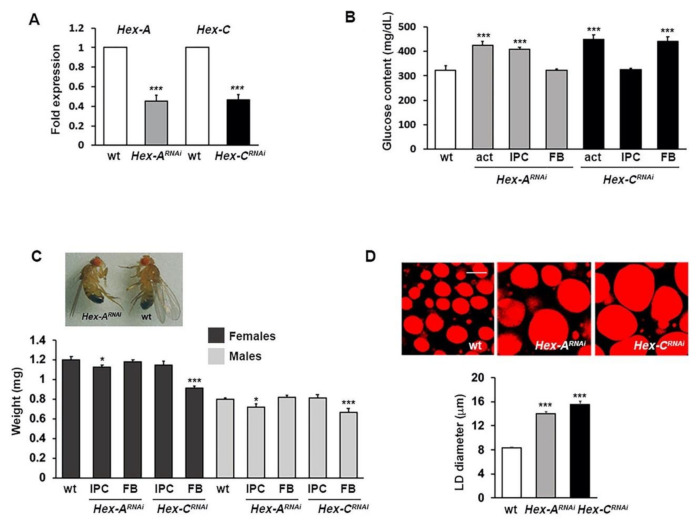
Silencing Hex-A and Hex-C results in diabetic phenotypes. (**A**) RT-qPCR analysis showing that both *Hex-A^RNAi^* and *Hex-C^RNAi^* individuals express significantly less transcript with respect to control. Fold changes in RNA levels relative to control were normalized to *α-tubulin* levels. Columns indicate the mean value ± SEM from three biological replicates. RNAs from *Hex-A^RNAi^* and *Hex-C^RNAi^* individuals were extracted from whole larvae and fat bodies, respectively. (**B**) Glucose content in larval hemolymph. *Hex-A* and *Hex-C* genes were ubiquitously silenced (act = actin-Gal4 driver) or specifically silenced either in insulin producing cells (IPC) or in the fat body (FB). Columns are the means of five independent sample measurements ± SEM. (**C**) *Hex-A^RNAi^* and *Hex-C^RNAi^* adults displayed a smaller body size compared to wild-type. Each column represents the mean weight (±SEM) of single flies. (**D**) Fat body larval lipid droplets (LD) stained with Nile Red. Scale bar 10 µm. Each column represents the mean diameter ± SEM calculated on about 100 LDs. *, *** Significantly different in the Student’s t test with *p* < 0.05 and *p* < 0.001, respectively.

To investigate the effects of Hex-A and Hex-C depletion, we silenced the corresponding genes by RNA interference (RNAi) using the *Hex-A v21054* and *Hex-C v35338* lines; individuals carrying the silenced genes will be hereafter referred to as *Hex-A^RNAi^* and *Hex-C^RNAi^*, respectively. Gene silencing, induced by the ubiquitous actin-Gal4 driver, reduced by 55% the amount of *Hex-A* and *Hex-C* transcripts and increased glucose levels in larval hemolymph, producing MODY-2 diabetic flies (Figure 1A,B). To further explore whether Hex-A is the counterpart of pancreatic GCK, we silenced this gene specifically in IPCs and measured the glucose content in *Hex-A^RNAi^* larval hemolymph. As shown in Figure 1B, silencing *Hex-A* in IPCs produced hyperglycemia (440 mg/dL vs. 320 mg/dL in the ctr), whereas the inactivation of this gene in the fat body resulted in a normal content of glucose. On the other hand, glycemia of *Hex-C^RNAi^* larvae was significantly higher than that of control when the gene was specifically silenced in the fat body (450 mg/dL) but not in IPCs (Figure 1B). These data were confirmed in two additional RNA lines (Hex-A RNAi line *# 35155* and Hex-C RNAi line *# 57404*) (Figure S1), overall suggesting that Hex-A and Hex-C enzymes may perform the roles played by mammalian GCK, in β-cells and hepatocytes, respectively.

We also found that *Hex-A^RNAi^* and *Hex-C^RNAi^* flies displayed other typical traits of diabetic flies besides hyperglycemia, such as decreased body size and impaired lipid metabolism [40,41]. In particular, the body size was significantly reduced in flies in which Hex-A depletion was induced in IPCs and in flies in which Hex-C was depleted in the fat body, but not vice versa Figure 1C and Figure S1).

We also evaluated the size of lipid droplets (LDs) in the fat bodies from *Hex-A^RNAi^* and *Hex-C^RNAi^* larvae. LDs are the major cellular organelles for lipid storage and their increased size has been associated with diabetes in flies [42,43]. We found that depletion of Hex-A or Hex-C resulted in a larger LD diameter compared to control larvae, suggesting that reduced activity of these enzymes could impair the lipid storage mechanism (Figure 1D).

### 2.2. Hex-A Is Involved in Insulin Secretion

**Figure 2:**
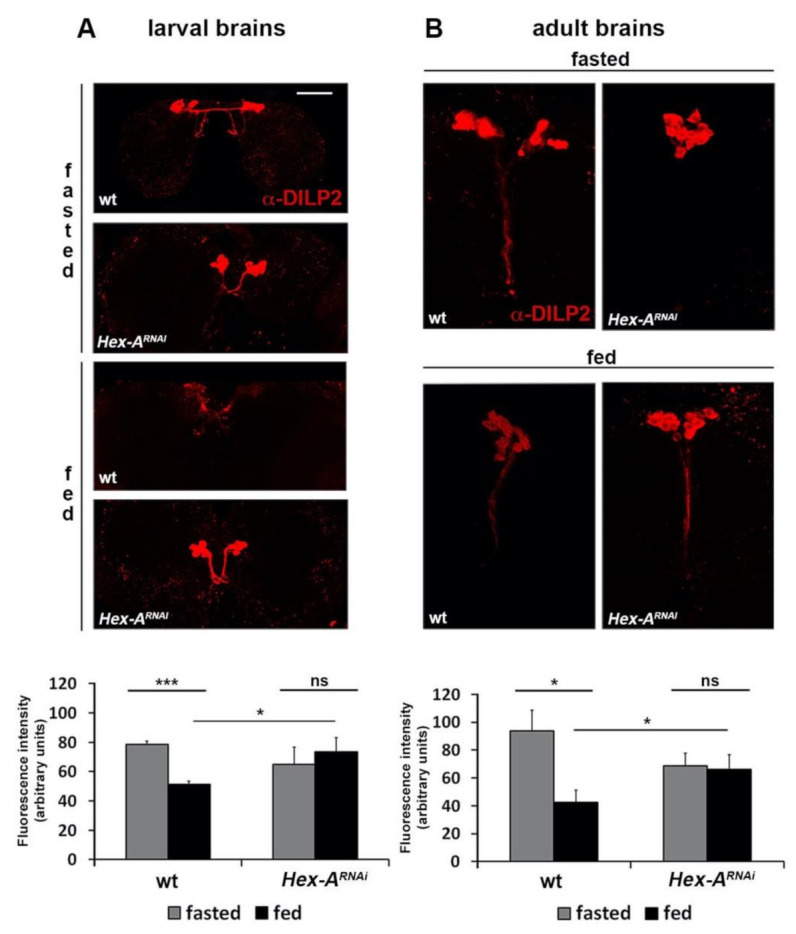
*Hex-A* functional inactivation impairs insulin release in larvae and adult flies. Confocal microscopy images showing larval (**A**) or adult (**B**) brains from fed or starved individuals immunostained with an anti-DILP2 (*Drosophila* insulin-like peptide 2) antibody. Brains isolated from fasted wild-type and *Hex-A^RNAi^* individuals show high levels of DILP2. After feeding, the DILP2 signal decreases in both larval and adult wild-type brains due to insulin secretion. In contrast, high DILP2 levels remain accumulated in Hex-A-depleted brains, suggesting impaired insulin secretion. Scale bar 50 µm. Bar graphs represent relative fluorescence ± SEM obtained processing each image through ImageJ software. *, *** Significantly different in the Student’s t test with *p* < 0.05 and *p* < 0.001, respectively; ns = not significant.

Mammalian GCK stimulates insulin secretion by promoting the opening of voltage-sensitive calcium channels, which in turn trigger hormone secretion [44]. To investigate whether *Hex-A* is involved in the same process, we fasted and re-fed for two hours *Hex-A^RNAi^* individuals and analyzed the insulin localization in larval and adult brains by immunofluorescence experiments. In wild-type brains from fasted larvae and adults there was a marked insulin accumulation that disappeared after feeding; conversely, the signal persisted after feeding in brains from *Hex-A^RNAi^* individuals, thus indicating that insulin delivery was reduced (Figure 2A,B). Taken together, these data confirm the involvement of *Hex-A* in the insulin secretion process and further support our hypothesis that *Hex-A* is the functional ortholog of pancreatic *GCK*.

### 2.3. Hex-A Regulates Hex-C Expression

**Figure 3:**
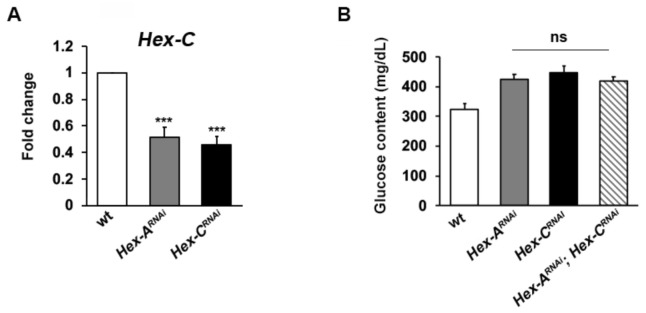
*Hex-A* regulates *Hex-C* expression. (**A**) RT-qPCR analysis showing that *Hex-C* expression is reduced in *Hex-A^RNAi^* larvae. Fold changes in RNA levels relative to control were normalized to *α-tubulin* levels. Columns indicate the mean value  ±  SEM from three biological replicates. (**B**) Glucose content in the hemolymph of *Hex-A^RNAi^* and *Hex-C^RNAi^* larvae. Note that the value found in double RNAi larvae is not different from that showed by *Hex-A^RNAi^*, indicating *Hex-A* over *Hex-C* epistasis. Columns are the means of five independent sample measurements ± SEM. *** Significantly different in the Student’s t test with *p* < 0.001; ns = not significant.

Insulin is one of the main inducers of the GCK hepatic isoform [14]; thus, we aimed to explore if a reduced expression of this gene could eventually regulate *Hex-C* expression, based on our finding that *Hex-A* is involved in insulin secretion. To answer this question, we reduced the expression of *Hex-A* by RNAi and we effectively observed a 50% drop of *Hex-C* expression through RT-qPCR experiments (Figure 3). This result was also confirmed using a different *Hex-A^RNAi^* line (Figure S2). Consistent with this result, the hemolymph of *Hex-A* and *Hex-C* double RNAi larvae showed a glucose content comparable to that of *Hex-A^RNAi^* individuals (Figure 3B), revealing *Hex-A* over *Hex-C* epistasis. Our finding strongly suggests that the mechanisms at the basis of GCK regulation are evolutionarily conserved, although in *Drosophila* the main GCK functions are redistributed between two genes.

### 2.4. The Depletion of Either Hex-A or Hex-C Causes Chromosome Aberrations

**Figure 4:**
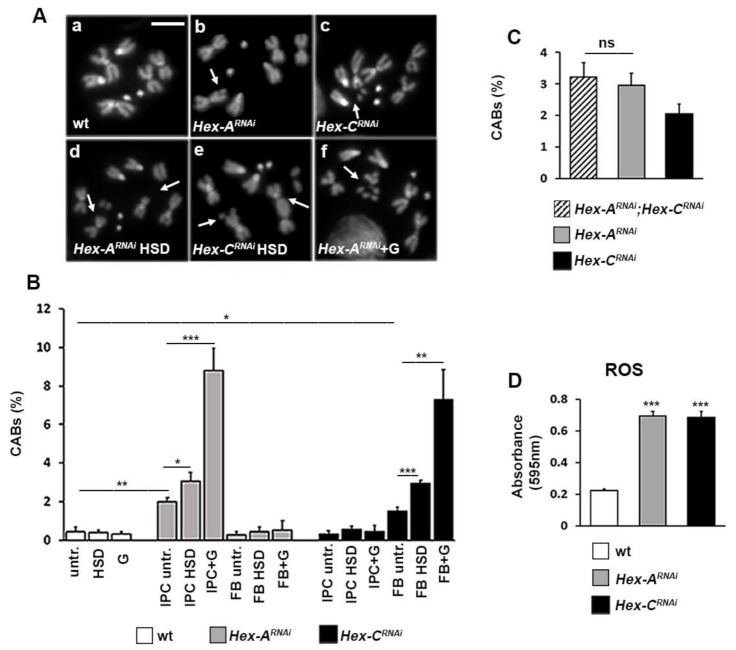
GCK depletion causes chromosome damage and reactive oxygen species (ROS) accumulation. (**A**) Examples of chromosome aberrations (CABs). (**a**) Wild-type female metaphase; (**b**) chromatid deletion of a major autosome, arrow; (**c**) isochromatid deletion of a major autosome, arrow; (**d**) two isochromatid deletions of major autosomes, arrows; (**e**) chromatid deletion of a major autosome and dicentric chromosome (autosome-autosome) accompanied by acentric fragments, arrows; and (**f**) fragmented major autosome, arrow. Scale bar, 5 μm. (**B**) Quantification of CABs. Each column represents the mean value ± SEM obtained by scoring at least 800 cells for each condition. IPC = insulin producing cells; FB = fat body; untr = untreated; G = 1% glucose; and HSD = high sugar diet (1 M sucrose). (**C**) CAB frequency in *Hex-A* and *Hex-C* double RNAi brains, which is not significantly different from that shown by *Hex-A^RNAi^* brains. (**D**) ROS quantification using nitroblue tetrazolium (NBT) assay in larval hemolymph of wild type and GCK-depleted larvae. Columns represent a mean value ± SEM of three different experiments. *, **, *** Significantly different in the Student’s t test with *p* < 0.05, *p* < 0.01 and *p* < 0.001, respectively; ns = not significant.

In order to understand the molecular and cellular mechanisms linking GCK to DNA damage, we found that tissue-specific silencing of either *Hex-A* or *Hex-C* genes caused chromosome aberrations (CABs) in larval brains with a frequency significantly higher than that of control (Figure 4A,B).

Rearing *Hex-A^RNAi^* and *Hex-C^RNAi^* larvae on a high sugar medium (sucrose 1 M vs. 0.15 M) enhanced CAB frequency (Figure 4B). Moreover, the incubation of brains isolated from the same larvae in 1% glucose increased the chromosome damage in contrast to that of control samples. As expected, the down regulation of *Hex-A* in the fat body as well as that of *Hex-C* in IPCs did not produce CABs, neither when brains were incubated in glucose nor when larvae were fed with a high sugar diet (HSD). These data were also confirmed by using different RNAi lines (Figure S3).

We then tested brains of *Hex-A* and *Hex-C* double RNAi larvae, finding a CAB frequency similar to that shown by *Hex-A^RNAi^* neuroblasts (Figure 4C). This result is consistent with the epistatic relationship already observed for glucose content in larval hemolymph (Figure 3B).

To better understand how CABs were generated, we evaluated the levels of oxidative stress by measuring ROS in Hex-A- and Hex-C-depleted larvae. It is known that hyperglycemia increases ROS production through different metabolic pathways, and ROS play a pivotal role in the development of diabetes complications [45,46]. Moreover, ROS can also attack DNA by producing chromosome damage, as testified by chromosome breakage found in patients with type 1 or 2 diabetes [47].

To detect the presence of ROS in *Hex-A^RNAi^* and *Hex-C^RNAi^* larval hemolymph, we used the nitroblue tetrazolium (NBT) assay, in which the interaction of NBT with superoxide generates a product (formazan) whose absorbance correlates with the amount of ROS [48]. As shown in Figure 4D, we found that ROS content in *Hex-A^RNAi^* as well as *Hex-C^RNAi^* larval hemolymph was higher compared to that of controls, suggesting that ROS can have a causal effect on CAB formation in GCK-depleted cells.

### 2.5. Hex-A- and Hex-C-Depleted Cells Accumulate Advanced Glycation End-Products (AGEs)

**Figure 5:**
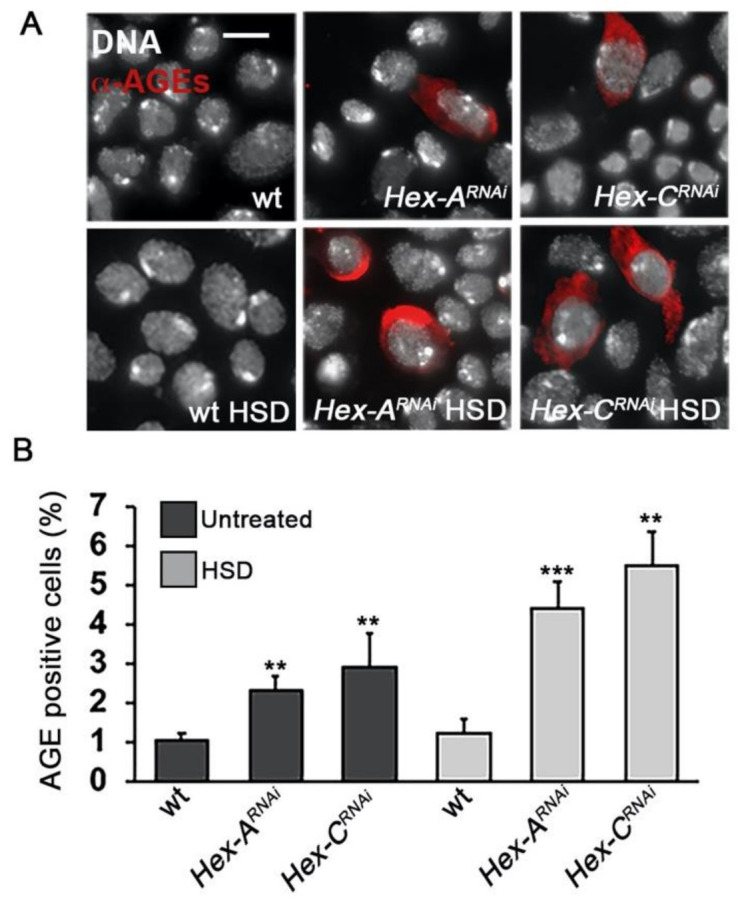
GCK-depleted larvae accumulate advanced glycation end-products (AGEs). (**A**) Examples of neuroblasts stained with a rabbit anti-human AGE antibody. Scale bar, 5 μm. (**B**) Frequencies of AGE-positive cells in wild-type (wt) and GCK-depleted brains from larvae grown on standard (untreated) or high sugar rich (HSD) medium. Bars represent the mean frequencies of AGE-positive cells ± SEM obtained by examining at least 1000 cells in 5 brains. **, *** Significantly different with respect to wild-type in the Student’s t test with *p* < 0.01 and *p* < 0.001, respectively.

One of the main metabolic routes that generate ROS in diabetes is the formation of advanced glycation end-products (AGEs). These toxic compounds are produced by hyperglycemia through non-enzymatic glycating reactions occurring between an excess of glucose and the amino groups of proteins and DNA [49]. It has been demonstrated that the reactions leading to AGEs are accompanied by an increased production of ROS [50]. In addition, we demonstrated that increased AGEs lead to the formation of chromosome damage in *Drosophila* [43,51]. To investigate whether the reduced GCK activity results in AGE accumulation, we performed immunostaining experiments using a human anti-AGE antibody. We found that larval neuroblasts from both *Hex-A^RNAi^* and *Hex-C^RNAi^* larvae displayed a significant percentage of cells positive to AGEs, that percentage increased when larvae were reared on an HSD medium (Figure 5). These results suggested that hyperglycemia resulting from impaired GCK function is responsible for AGE accumulation in *Drosophila*, which in turn may cause CABs through an increased ROS production.

### 2.6. Vitamin B6 Prevents Chromosome Damage in Drosophila MODY-2

We previously demonstrated that reduced levels of vitamin B6 (pyridoxal 5′-phosphate, PLP) combined with hyperglycemia cause a large amount of DNA damage through the AGE pathway in *Drosophila* and human cells [51,52]. PLP, besides working as a cofactor in more than 150 metabolic reactions, plays a pivotal role as an antioxidant molecule, counteracting the formation of both ROS and AGEs [53]. Given this premise, we asked whether PLP supplementation was able to rescue CABs in brains from *Hex-A^RNAi^* and *Hex-C^RNAi^* larvae and, on the other hand, whether the strong PLP inhibitor 4-deoxypiridoxine (4-DP) could increase DNA damage in our MODY-2 fly models.

**Figure 6:**
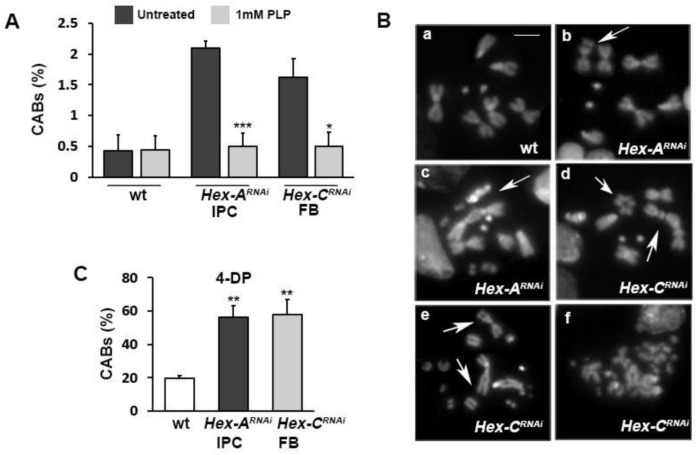
CABs are rescued by PLP and enhanced by 4-DP in *Hex-A^RNAi^* and *Hex-C^RNAi^* neuroblasts. (**A**) Percentage of CABs in neuroblasts from larvae grown in either standard or PLP-supplemented (1 mM) medium. Each column represents the mean value ± SEM obtained by scoring at least 800 cells in six brains. Statistical significance is expressed with respect to the untreated condition. (**B**) Examples of chromosome rearrangements induced by the PLP inhibitor 4-DP (2 mM) (**a**) Wild type female metaphase; (**b**) isochromatid deletion of a major autosome, arrow; (**c**,**d**,**e**) metaphases showing extensive chromosome fragmentation arrowed; (**f**) metaphase with highly fragmented chromosomes. Scale bar, 5 μm. (**C**) Percentage of CABs in neuroblasts from 4-DP-treated larvae. *, **, *** Significantly different in the Student’s t test with *p* < 0.05, *p* < 0.01 and *p* < 0.001, respectively.

As shown in Figure 6A, whereas PLP treatment completely rescued CABs by restoring control values, 4-DP treatment enhanced CAB frequency in both *Hex-A^RNAi^* and *Hex-C^RNAi^* larvae, producing a higher and more complex level of breaks with respect to control samples (Figure 6B,C and Figure S4).

Taken together, these data support the model that CABs are induced by ROS and in turn generated through the AGE pathway. In addition, they suggest that the antioxidant properties of vitamin B6 may reduce the risk of DNA damage in MODY-2.

## 3. Discussion

In the past decade, *Drosophila* has emerged as a valid model organism for studies concerning metabolic diseases, including diabetes [54]. Major metabolic pathways and physiological responses have proven to be conserved in flies, though sometimes when functions are carried out by a single mammalian gene, they are performed by multiple *Drosophila* orthologs. For instance, *Drosophila* insulin is encoded by eight different genes (DILP 1-8) that overall play the roles performed by the human single peptide [55].

In the present work, we characterized the GCK role in *Drosophila*, in particular focusing on the effects of its depletion on genome integrity.

Differently from higher organisms in which GCK function is played by a single gene, the *Drosophila* genome harbors four *GCK* orthologs, but only *Hex-C* has been in part characterized [39]. Here we showed, for the first time, that the *GCK* ortholog *Hex-A* is specifically expressed in IPCs, and it is involved in insulin secretion. This result suggests that *Drosophila Hex-A* and *Hex-C* genes play the same role as the human pancreatic and hepatic isoforms, respectively. Interestingly, we found that *Hex-A* is required for *Hex-C* expression. Given that *Hex-A* is involved in insulin secretion, our finding fits perfectly with the knowledge that insulin is a crucial regulator of GCK hepatic expression in mammals. Several transcription factors, including hypoxia-inducible factor 1 alpha (HIF1α) and sterol regulatory element–binding protein-1c (SREBP-1c), have been identified as possible insulin mediators [14]. Due to their evolutionary conservation, we expect that these factors may work as insulin downstream effectors in *Drosophila* too. Further studies are required to investigate this relationship.

Given that MODY-2 is caused by heterozygous inactivating *GCK* mutations [18], RNAi-induced silencing of either *Hex-A* or *Hex-C* genes represents a useful strategy to generate MODY-2 flies, reducing the amount of transcripts by about 55%. However, given the epistatic relation linking *Hex-A* to *Hex-C*, it is conceivable that *Hex-A^RNAi^* flies are indeed the closest model of *Drosophila* MODY-2, as they recapitulate all the hallmarks associated with GCK-depletion, including insulin secretion defects.

In this work, we provided evidence that reduced levels of either *Hex-A* or *Hex-C* cause chromosome damage. We also showed that the depletion of either *Hex-A* or *Hex-C* result in high levels of ROS and AGE accumulation. It is known that persistent hyperglycemia leads to oxidative stress, producing ROS through different metabolic routes. Among these, AGE formation has an important role, taking into account the large body of evidence linking AGEs to micro- and macrovascular diabetic complications [56]. Based on these considerations and on our data, we hypothesized that in GCK-MODY flies, CABs are induced by ROS produced during AGE metabolism. The evidence that hyperglycemia can induce chromosome breakage is sustained by the presence of CABs and micronuclei in patients affected by type 1 or type 2 diabetes [57,58,59]. Moreover, in our previous studies we found that AGE accumulation is largely responsible for CAB formation in two different type 2 diabetic fly models, thus confirming our hypothesis and the validity of a *Drosophila* model in this context [43]. Our hypothesis that chromosome damage is triggered by AGE-induced ROS is further supported by CAB rescue obtained with vitamin B6 (PLP) antioxidant treatment in our MODY-2 flies. This result is in line with the capability of PLP to counteract ROS [53] and reduce AGE formation by sequestering 3-deoxyglucosone (3-DG), an intermediate product of the AGE route [60]. In parallel, we showed that Hex-A- as well as Hex-C-depleted neuroblasts were more sensitive to 4-DP (a PLP inhibitor) treatment with respect to controls. 4-DP produced not only a high frequency of CABs (60% vs. 20% in controls) but also a more complex pattern of breakage. Similar results were found in type 2 diabetes fly models [43], thus suggesting that a low vitamin B6 level can also be genotoxic in MODY-2 diabetes.

To date, the direct impact of hyperglycemia on DNA damage has never been studied in GCK-MODY; however, a recent work showed that reduced levels of GCK had an impact on telomere metabolism. An inverse correlation between telomere length and fasting glycemia as well as between telomere length and glycated hemoglobin levels have been reported in patients with MODY-2 [61]. It has been proposed that telomere shortening found in patients with type 1 or type 2 diabetes is caused by oxidative stress [62,63]; thus, the finding of reduced telomere length in MODY-2 cells gives a robust support to our hypothesis that oxidative stress can threaten genome integrity in GCK-depleted cells.

The presence of ROS and CABs in MODY-2 flies seems to be in apparent contrast with the low prevalence of oxidative stress-induced complications in patients with GCK-MODY. One hypothesis to explain this discrepancy is that MODY-2 complications may have been underestimated in the population. In fact, a systematic assessment of MODY-2 complications has been performed only once, in a group of 50-year-old patients, revealing a 30% prevalence of retinopathy or significant occurrence of other diseases [26]; previous studies revealing only isolated cases of complications have been performed on young people or in small groups of patients [18,25,64]. In addition, many MODY-2 cases are often misclassified as type 1 or type 2 diabetes, with the consequence that possible complications are wrongly attributed to these forms of diabetes. Another and more convincing explanation concerns the genetic mechanisms underlying MODY-2. It has been observed that regardless the severity of *GCK* mutations, clinical phenotypes shown by patients with GCK-MODY are relatively similar. To explain this, it has been proposed that in MODY-2 β-cells, the wild type *GCK* allele is overexpressed to compensate for the mutant allele, thus preventing a severe drop in GCK activity [65]. Our MODY-2 flies could, thus, display more severe phenotypes because the allele compensation does not occur, given that the expression of either *Hex-A* or *Hex-C* is down-regulated by RNA interference. In strong agreement with this hypothesis, it has been recently demonstrated that a MODY-2 rabbit model, where GCK activity was reduced by 50% due to a homozygous (36 bp) deletion, displayed severe diabetes complications, such as kidney diseases, feet ulcerations, and osteoporosis [66].

In conclusion, our studies showed for the first time that a MODY-2 fly model can be generated by the targeted disruption of the Drosophila *Hex-A* gene. Moreover, we provided evidence that reduced GCK levels produce oxidative stress and chromosome damage in *Drosophila*. However, considering that MODY-2 has been associated to complications only in a few cases, our data suggest that these patients need to be evaluated case by case and eventually treated when diabetes is accompanied by other risk factors threatening genome integrity or reducing antioxidant defenses. Given the antioxidant role of vitamin B6 in diabetes [47], a dietary PLP-enriched supplement may represent a new strategy to prevent or reduce oxidative stress.

## 4. Materials and Methods

### 4.1. Drosophila Stocks and Crosses

*Hex-A^v21054^ and Hex-C^v35338^* lines were obtained from the Vienna Drosophila Resource Center (VDRC) stock center. *Hex-A^35155^*, *Hex-C^v57404^*, *ppl*-Gal-4 (fat body driver), and *w*; *Pw [+mC] = Ilp2-GAL4.R2/CyO* (IPC driver) were obtained by Bloomington Drosophila Stock Center (BDSC). The *Oregon R* strain was used as the wild-type control. All stocks were maintained and crosses were made at 25 °C on standard or supplemented medium (see below). To generate individuals carrying both *Hex-A* (v21054) and *Hex-C* (v35338) RNAi constructs, we crossed *Hex-A^RNAi^/Hex-A^RNAi^; MKRS/TM6B* males to *CyGFP/Sco*; *act-Gal4/TM6B* females. *Hex-A^RNAi^/CyGFP*; *act-Gal4/TM6B* male progeny were then crossed to *Hex-C^RNAi^/Hex-C^RNAi^* females to obtain *Hex-A^RNAi^*/*Hex-C^RNA^*^i^; *act-Gal4* larvae that were recognized for their non-GFP and non-*Tubby* phenotype. The used balancers and genetic markers are described in detail in FlyBase (http://flybase.bio.indiana.edu/).

### 4.2. Fly Food Recipes

All flies, from embryo stage, were raised at 25 °C on standard food or on a sugar-rich medium (HSD), which is a standard food with an increased sucrose concentration. Food (per 100 mL) contained agar (0.68 g), yeast (6.52 g), flour (3 g), propionic acid (600 μL), and sucrose (5.13 g = 0.15 M for standard food; 34.2 g = 1.0 M for high sugar diet, HSD). To test the effects of vitamin B6 supplementation, PLP was dissolved in standard food at 1 mM concentration. Fasting was achieved by using 1% agar as a medium; adults were fasted 24 h, while third-instar larvae were fasted for 8 h.

### 4.3. Chromosome Cytology

Colchicine-treated *Drosophila* metaphase chromosome preparations for CAB scoring were obtained as previously described [67]. For AGE detection, brain preparations from third instar larvae were carried out according to Bonaccorsi et al. [68]. Preparations were rinsed in PBS 0.1% Triton (PBST), incubated overnight at 4 °C with a rabbit anti-human AGE antibody (1:200 in PBST; ab23722, Abcam, Cambridge, UK), rinsed in PBST, and then incubated for 1 h at room temperature with the Alexa-Fluor-555-conjugated anti-rabbit secondary antibody (1:300 in PBST; Molecular Probes). All fixed preparations were mounted in Vectashield H-1200 with 4,6-diamidino-2-phenylindole (DAPI) (Vector Laboratories, Burlingame, CA, USA) to stain the DNA. To quantify AGE-positive neuroblasts, at least 1000 cells were analyzed for each genotype. All cytological preparations were examined with a Carl Zeiss (Thornwood, NY, USA) Axioplan fluorescence microscope, equipped with an HBO100W mercury lamp and a cooled charged-coupled device (CCD camera; Teledyne Photometrics, Tucson, AZ, USA).

### 4.4. Immunostaining of Larval and Adult Drosophila Brains

To evaluate insulin secretion, immunofluorescence on fly larval and adult brains was performed according to Wu and Luo [69] with slight modifications. Brains were dissected in a 0.1 M PB solution (100 mM Na_2_HPO_4_/NaH_2_PO_4_, pH 7.2), washed in a 0.1 M PB 0.2% Triton (PBT) solution, and then fixed for 30 min in a 4% formaldehyde PBT solution. Blocking was done in a 5% NGS (Normal Goat Serum, Merck, Darmstatd, Germany) PBT solution for at least 40 min. Primary and secondary antibodies were diluted in a 5% NGS PBT solution, incubated for two nights at 4 °C on a rotating wheel, and then mounted in Fluoromount medium (Merck, Darmstatd, Germany). The primary antibody used was rat anti-DILP2 (1:50, a gift from Pierre Leopold, Curie Institut, Paris, France). The fluorescent-labeled secondary antibody raised in goat was Cyanine Cy™3-conjugated anti-rat (1:300, Jackson ImmunoResearch Europe, Ely, UK). Immunofluorescence analysis was performed through a confocal laser scanning microscope (LSM800, Zeiss, Jena, Germany) equipped with four laser lines: 405 nm, 488 nm, 561 nm, and 639 nm. The brightness and contrast of the digital images were adjusted using Zeiss Zen software 3.0 blue edition (Zeiss, Jena, Germany) and Adobe Photoshop CS6 (Adobe, San Jose, CA, USA).

### 4.5. Treatments of Larvae and Isolated Brains

To evaluate the effects of PLP inhibitor 4-deoxypyridoxine (4-DP) on CABs, 4-DP 2 mM was added to medium containing first and second instar larvae. Three days later, third instar larvae were dissected and their brains were fixed.

To test the effects of glucose on CABs, brains were dissected from third instar larvae and incubated in 2 mL of saline supplemented with 10% fetal bovine serum (FBS, Corning, New York, NY, USA) for 4 h with or without addition of 1% glucose. One hour before fixation, brains were treated with colchicine and fixed according to Gatti and Goldberg [67].

### 4.6. Glucose Measurement and Weight Analysis

Glucose concentration in third instar larvae hemolymph was measured using the Infinity Glucose Hexokinase reagent (Thermo Scientific, Waltham, MA, USA). Hemolymph collection and glucose measurement were done as described in Marzio et al. [51].

For weight analysis, 5–6 samples of 15 flies each were weighted with a precision weight scale (Gibertini E42; range 0.1 mg–120 g). Flies were reared under the same growth conditions and were age-matched (2 days old) before weighing.

### 4.7. Lipid Droplet Measurement

To stain lipid droplets (LDs), fat bodies of third instar larvae were dissected in PBS. Fat bodies were fixed in 4% formaldehyde for 30 min at room temperature. Tissues were then rinsed twice in PBS and incubated for 30 min in a 0.5 μg/mL Nile Red PBS 1X solution. After being rinsed twice with PBS, fat bodies were mounted in Vectashield Antifade Mounting Medium (Vector Laboratories, Romford, UK) and analyzed to a confocal microscope Zeiss LSM 780. Slides were imaged at 63X magnification. The ImageJ software (version 1.51j, NIH, Bethesda, MD, USA) was used to quantify LD size.

### 4.8. Nitroblue Tetrazolium (NBT) Assay

A total of 5 µL of hemolymph extracted from 15 third instar larvae were mixed with 10 μL of PBS 1X and an equal volume of 0.16 mM NBT. Samples were left for 1 h in the dark at room temperature. The reaction was stopped by adding 30 μL of 100% glacial acetic acid. Samples were centrifuged at maximum speed for 1 min and the absorbance was measured at 595 nm.

### 4.9. RNA Extraction, Reverse Transcription, and RT-qPCR

RNA was extracted using the NucleoSpin RNA kit (Macherey and Nagel, Bethlehem, PA, USA) from three biological replicates, each made of 20 third instar larvae or 50 larval fat bodies. RNA concentration and quality were evaluated by measuring in 0.1 N NaOH the OD at 260 nm and the ratio at 260/280 nm, respectively, and by electrophoresis on 1.2% agarose gels. Reverse transcription of DNase-treated RNAs (1 μg) was carried out using the OneScript^®^ Plus cDNA Synthesis Kit (ABM Good, Richmond, BC, Canada) with the random primers provided in the kit. RT-qPCR was performed on a CFX Connect Real Time PCR system (Bio-Rad, Hercules, CA, USA) with a two-step reaction using SYBR green ExcelTaq™ Master Mix (SMOBIO, Hsinchu City, Taiwan) and the oligonucleotides reported below. The relative expression of each target gene was determined by the Pfaffl method using the α-tubulin as the normalizer. The fold induction resulting from the different pairs of samples was averaged and the *p* value was calculated using the Student’s *t*-test.

*Hex-A*_for CAATGTGCGGTACATCTGCG

*Hex-A*_rev TTGGGATGGAAGCGGTACAC

*Hex-C*_for GTCGCTTTTGCCTGGAAGTG

*Hex-C*_rev TGGTGACCTTTCAGCGAGAC

*α-tubulin_* for TGTCGCGTGTGAAACACTTC

*α-tubulin_* rev AGCAGGCGTTTCCAATCTG

## Supplementary Materials

Supplementary Materials can be found at https://www.mdpi.com/1422-0067/22/2/918/s1: Figure S1: GCK depletion in Hex-A^v35155^ or Hex-C^v57404^ RNAi flies results in diabetic phenotypes. Figure S2: Hex-A regulates Hex-C expression. Figure S3: GCK depletion causes CABs in brains from Hex-A^v35155^ and Hex-C^v57404^ RNAi larvae. Figure S4: CABs are rescued by PLP and enhanced by 4-DP in Hex-A^v35155^ and Hex-C^v57404^ RNAi neuroblasts.

## Abbreviations

4-DP	4-deoxypiridoxine
AGEs	Advanced glycation end-products
CABs	Chromosome aberrations
DILP2	Drosophila insulin-like protein 2
GCK	Glucokinase
GKRP	Glucokinase regulatory protein
FB	Fat body
HSD	High sugar diet
IPCs	Insulin producing cells
LDs	Lipid droplets
MODY	Maturity-onset diabetes of the young
NBT	Nitroblue tetrazolium
PFK2/FBP2	6-phosphofructo-2-kinase/fructose 2,6-bisphosphatase
PLP	Pyridoxal 5′-phosphate
ROS	Reactive oxygen species
