# Supplementary material for: Functional Inactivation of Drosophila GCK Orthologs Causes Genomic Instability and Oxidative Stress in a Fly Model of MODY-2
Source: Int J Mol Sci. 2021 Jan 18;22(2):918. doi: 10.3390/ijms22020918 (PMC7831483; doi:10.3390/ijms22020918)
Supplement: Supplementary file 1 [file ijms-22-00918-s001.pdf]

Supplementary figures

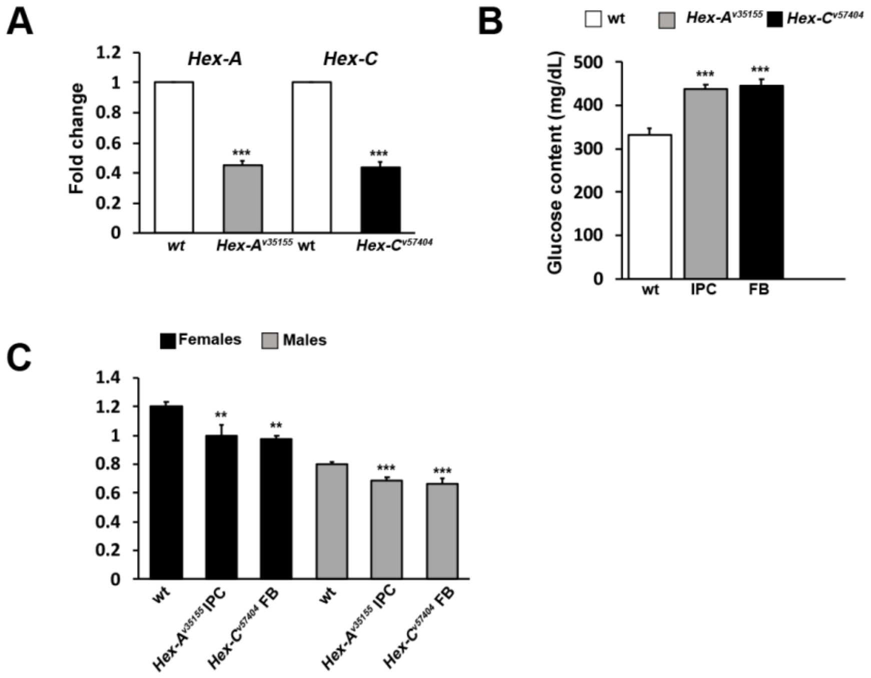

Figure S1. *GCK depletion in Hex-A*^{v35155} *or Hex-C* ^{v57404} *RNAi flies results in diabetic phenotypes*. **(A)** RT-qPCR analysis showing that both *Hex-A*^{RNAi} and *Hex-C*^{RNAi} individuals express significantly less transcript with respect to control. Fold changes in RNA levels relative to control were normalized to *α-tubulin* levels. Columns indicate the mean value ± SEM from three biological replicates. RNAs from *Hex-A*^{RNAi} and *Hex-C*^{RNAi} individuals were extracted from whole larvae and fat bodies respectively. **(B)** Glucose content in larval hemolymph. *Hex-A* or *Hex-C* genes have been silenced in insulin producing cells (IPC) or in fat body (FB). Columns are the means of five independent sample measurements ± SEM. **(C)** *Hex-A*^{RNAi} and *Hex-C*^{RNAi} adults display a smaller body size compared to wild-type. Each column represents the mean weight (± SEM) of single flies. *,*** Significantly different in the Student's t test with $p < 0.05$ and $p < 0.001$ respectively.

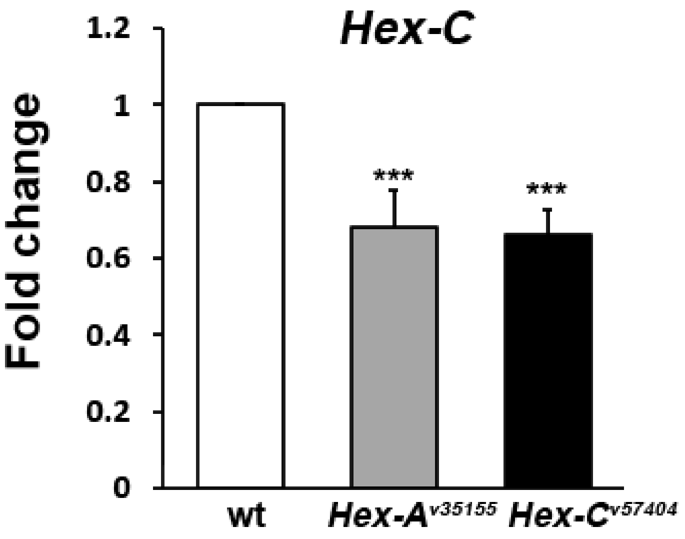

Figure S2. *Hex-A regulates Hex-C expression.* RT-qPCR analysis showing that *Hex-C* expression is reduced in *Hex-A*[RNAi] larvae from v35155 RNAi line. Fold changes in RNA levels relative to control were normalized to *α-tubulin* levels. Columns indicate the mean value ± SEM from three biological replicates. *** Significantly different in the Student's t test with $p < 0.001$.

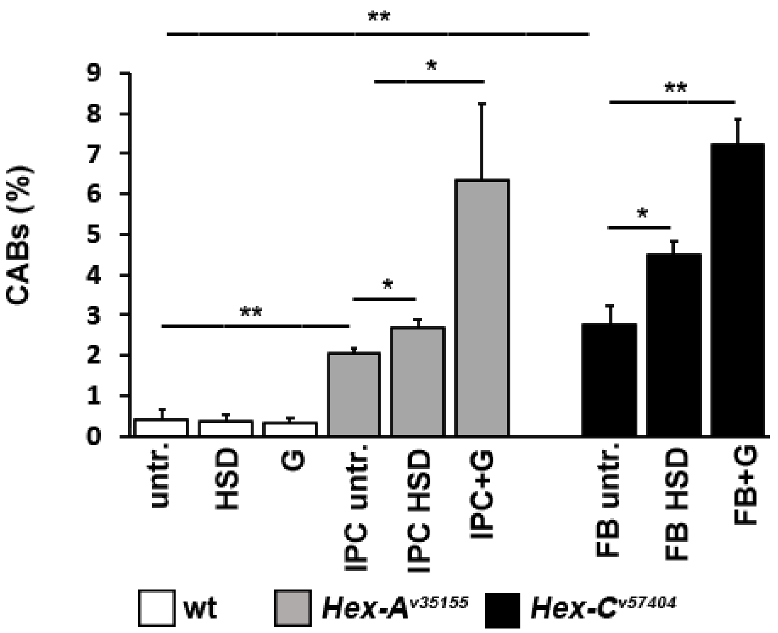

Figure S3. *GCK depletion causes CABs in brains from Hex-A*[v35155] *and Hex-C* [v57404] *RNAi larvae.* Percentage of CABs in brains from larvae grown in standard or HSD medium and in brains treated with 1% glucose (G). Each column represents the mean value ± SEM obtained by scoring at least 800 cells for each condition. *,** Significantly different in the Student's t test with $p < 0.05$ and $p<0.01$ respectively.

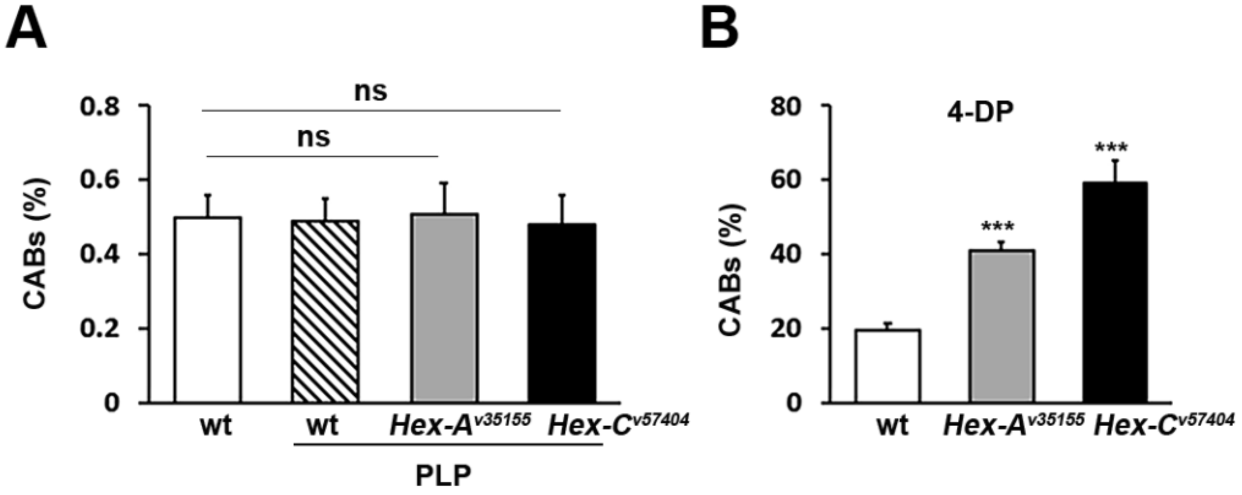

Figure S4. *CABs are rescued by PLP and enhanced by 4-DP in Hex-A*^{v35155} *and Hex-C*^{v57404} *RNAi neuroblasts*. **(A)** Percentage of CABs in neuroblasts from larvae grown in PLP (1mM) supplemented medium. Each column represents the mean value ± SEM obtained by scoring at least 800 cells in six brains. **(B)** Percentage of CABs in neuroblasts from 2mM 4-DP treated larvae. *** Significantly different in the Student's t test with $p < 0.001$; ns=no significant
